# Synergy of Glutamatergic and Cholinergic Modulation Induces Plateau Potentials in Hippocampal OLM Interneurons

**DOI:** 10.3389/fncel.2019.00508

**Published:** 2019-11-12

**Authors:** Nicholas Hagger-Vaughan, Johan F. Storm

**Affiliations:** Brain Signaling Laboratory, Section for Physiology, Institute of Basic Medical Sciences, University of Oslo, Oslo, Norway

**Keywords:** interneuron, plateau potential, OLM cells, synergy, mGluR, mAChR, TRP channel

## Abstract

Oriens-lacunosum moleculare (OLM) cells are hippocampal inhibitory interneurons that are implicated in the regulation of information flow in the CA1 circuit, inhibiting cortical inputs to distal pyramidal cell dendrites, whilst disinhibiting CA3 inputs to pyramidal cells. OLM cells express metabotropic cholinergic (mAChR) and glutamatergic (mGluR) receptors, so modulation of these cells *via* these receptors may contribute to switching between functional modes of the hippocampus. Using a transgenic mouse line to identify OLM cells, we found that both mAChR and mGluR activation caused the cells to exhibit long-lasting depolarizing plateau potentials following evoked spike trains. Both mAChR- and mGluR-induced plateau potentials were eliminated by blocking transient receptor potential (TRP) channels, and were dependent on intracellular calcium concentration and calcium entry. Pharmacological tests indicated that Group I mGluRs are responsible for the glutamatergic induction of plateaus. There was also a pronounced synergy between the cholinergic and glutamatergic modulation, plateau potentials being generated by agonists applied together at concentrations too low to elicit any change when applied individually. This synergy could enable OLM cells to function as coincidence detectors of different neuromodulatory systems, leading to their enhanced and prolonged activation and a functional change in information flow within the hippocampus.

## Introduction

Hippocampal CA1 pyramidal cells receive two different streams of input: one from the entorhinal cortex (EC) conveying a representation of the present external world, and another from the hippocampal CA3 region conveying mnemonic information from past experiences (Vago et al., 2007).

Oriens-lacunosum moleculare (OLM) cells are inhibitory interneurons located in the CA1 region, and their activation has been implicated in regulating information flow in the hippocampus, facilitating CA3 input whilst weakening the EC input (Leão et al., 2012).

The CA1 network shifts rhythmically between responding to one or the other stream of input (Colgin et al., 2009). Modulation of OLM cell activity may be a key contributor to this switching between functional modes in the hippocampus. However, relatively little is known about the mechanisms underlying the neuromodulation of OLM cells.

OLM cells somata are located in the oriens-alveus layer of the CA1, with dendrites that branch within the oriens layer where they receive glutamatergic input from local CA1 pyramidal cells (Blasco-Ibáñez and Freund, 1995) and cholinergic input from the medial septum (Leão et al., 2012). Their axons project to the lacunosum-moleculare layer, where they mediate pre- and post-synaptic inhibition of input to the distal apical dendritic tufts of hippocampal pyramidal cells and disinhibition to pyramidal cell proximal dendrites (McBain et al., 1994; Sik et al., 1995; Leão et al., 2012).

OLM cells are characterized by spontaneous spiking in slice preparations (Leão et al., 2012) as well as in anesthetized (Klausberger et al., 2003) and awake (Varga et al., 2012) animals, which implies that OLM cells provide their targets with a constitutive tonic inhibition (Perez et al., 2001; Lamsa et al., 2007).

OLM cells express both metabotropic cholinergic (mAChR; van Hooft et al., 2000; Ferraguti et al., 2004; Le Vasseur et al., 2008) and metabotropic glutamatergic (mGluR; Lawrence et al., 2006; Le Duigou et al., 2015) receptors. Metabotropic glutamatergic and muscarinic effects have previously been reported in relation to LTP induction in OLM cells (Le Vasseur et al., 2008; Le Duigou et al., 2015). However, the effects of mGluR and mAChR modulation on the intrinsic properties of the cells and their responses to input (which may underlie these changes in plasticity) have not been fully addressed. Of particular interest is the convergence of modulatory pathways in regulating neuronal behavior, as shown in CA1 pyramidal cells (Park and Spruston, 2012). Synergy can provide an opportunity for selectively altering the activity of specific neuron classes by the combined activation of two neuromodulatory systems, either by altering intrinsic neuronal properties and immediate response to repeated action potential firing (Park and Spruston, 2012), or by inducing plastic changes which alter their response to synaptic input (Le Duigou et al., 2015). This is likely to occur often during arousal and wakefulness in behaving animals, when multiple neurotransmitter systems are active simultaneously (Watson et al., 2010), but this has so far not been assessed in interneurons.

Plateau potentials are extended periods of depolarization following in the wake of action potentials generated by excitatory synaptic input, often caused by the opening of calcium or other cation channels (Milojkovic et al., 2005). Several different functions have been ascribed to plateau potentials. They have been proposed as a mechanism for working memory (Egorov et al., 2002; Hasselmo and Stern, 2006), and are associated with LTP induction (Gambino et al., 2014). The plateaus can extend the period of spiking in response to excitatory input beyond the decay of the initial synaptic barrage. This creates an extended window where plasticity may be induced in a Hebbian (Gambino et al., 2014) or non-Hebbian (Bittner et al., 2017) manner, at behaviorally relevant time scales (Bittner et al., 2017). Both mAChRs and mGluRs have been shown to cause depolarizing spike afterpotentials (Klink and Alonso, 1997; Young et al., 2004) and spontaneous and persistent firing (Azouz et al., 1994; Yoshida et al., 2008), features associated with plateau potentials in a range of cell types across several animal species.

Convergent evidence indicates that mGluRs are highly important for circuit functions in regards to conveying both semantic and modulatory information (Sherman, 2014). Furthermore, both Group I and Group II mGluRs can be activated by only brief trains of 2–3 presynaptic spikes (Viaene et al., 2013), indicating that only sparse activation is needed to elicit metabotropic effects. Since the glutamatergic synapses onto OLM cells are facilitatory (Silberberg and Markram, 2007; Kim, 2014), they probably belong to the so-called “Class 2” inputs (Viaene et al., 2013), which are known to activate mGluRs in addition to ionotropic GluRs (iGluRs), suggesting that metabotropic activation is likely to be a regular feature at excitatory synapses on OLM cells.

Since OLM cells are probably crucially involved in several functions that are subject to neuromodulation, and these cells are themselves equipped with a variety of receptors and ion channel types that can mediate neuromodulation, it is highly relevant to explore this dimension of their functional repertoires. However, there have to date been only a few studies of neuromodulation in these cell types (McBain et al., 1994; Le Vasseur et al., 2008; Le Duigou et al., 2015). This study aims to fill some of these gaps, by focusing on the neuromodulatory effects of two of the main neurotransmitters in the brain: glutamate and acetylcholine—alone and in combination.

To study glutamatergic and muscarinic metabotropic modulation in hippocampal OLM cells we used a *Chrna2*-cre transgenic mouse line, because the *Chrna2*-cre gene has been shown to be specifically expressed in OLM cells (Mikulovic et al., 2015). We show that these genetically identified OLM cells exhibit plateau potentials in response to both mAChR and mGluR activation. Pharmacological tests indicated that these responses were dependent on non-specific cation channels of the transient receptor potential (TRP) family, and the glutamatergic plateaus were mediated by the group I mGluRs. mGluR activation also increased the spontaneous firing rate of the cells and increased excitability in response to the current input. Our results suggest that the metabotropic receptors’ activation of OLM cells dramatically increases their activity in response to depolarizing input and allows ongoing action potential firing to continue beyond the offset of these inputs. We also suggest that intracellular signaling pathways of both mAChRs and mGluRs are convergent and that simultaneous activation elicits plateau potentials in a synergistic, supra-linear manner.

## Materials and Methods

### Ethical Approval

All animal procedures were approved by the responsible veterinarian of the institute, in accordance with the statute regulating animal experimentation (Norwegian Ministry of Agriculture, 1996).

### Animals

*Chrna2-cre* transgenic C57BL6 mice were donated for breeding by the Kullander group and have been previously described (Leão et al., 2012). *Gt(ROSA)26Sor*^tm14(CAG-tdTomato)Hze^ (*R26*^tom^) transgenic mice were obtained from Jackson Laboratories. *Chrna2-cre* and *R26*^tom^ lines were crossbred to give a line expressing RFP variant tdTomato under control of the *Chrna2* gene (*Chrna2-cre; R26*^tom^).

### Hippocampal Slice Preparation

Horizontal hippocampal slices were obtained from both male and female *Chrna2-cre; R26* mice between 4 and 8 weeks of age. Mice were anesthetized with isoflurane inhalation and decapitated, and the brain was removed quickly into ice-cold sucrose-based artificial cerebrospinal fluid (aCSF) containing (in mM): 1.25 NaCl, 1.25 KCl, 1.25 NaH_2_PO_4_, 7 MgCl_2_, 0.5 CaCl_2_, 16 glucose, 75 sucrose, 25 NaHCO_3_) saturated with 95% O_2_–5% CO_2_. 350 μm slices were cut using a Leica VT1200 vibratome (Leica Microsystems; Wetzlar, Germany) and incubated for 30 min at 35°C in aCSF containing (in mM): 125 NaCl, 2.5 KCl, 1.25 NaH_2_PO_4_, 1.4 MgCl_2_, 1.6 CaCl_2_, 16 glucose, 25 NaHCO_3_) saturated with 95% O_2_–5% CO_2_. After incubation slices were kept at room temperature (~20°C) until use.

### Electrophysiology

Whole-cell and cell-attached patch-clamp recordings were obtained using visual guidance from IR-DIC optics (BX51; Olympus, Tokyo, Japan) from the somata of OLM cells identified by widefield fluorescence microscopy.

Slices were maintained at 32 ± 0.5°C and superfused with aCSF containing the AMPA, NMDA and GABA_A_ receptor blockers 6,7-dinitroquinoxaline-2,3-dione (DNQX; 10 μM), DL-2-Amino-5-phosphonopentanoic acid (DL-AP5; 50 μM) and SR 95531 (gabazine; 5 μM) respectively, in order to block both excitatory and inhibitory synaptic transmission. Patch-clamp pipettes (5–7 MΩ) were pulled from borosilicate glass tubing (outer diameter 1.5 mm, inner diameter 0.86 mm, with filament; Sutter Instruments, Novato, CA, USA) and filled with a solution containing (in mM): 120 potassium gluconate, 20 KCl, 5 phosphocreatine disodium salt, 4 MgATP, 0.4 NaGTP, 10 HEPES and 0.1 EGTA. The pH of the intracellular medium was adjusted to 7.2 with KOH, and osmolarity was between 280 and 290 mOsmol^−1^. Recordings were made using a Multiclamp 700A patch-clamp amplifier (Molecular Devices; Sunnyvale, CA, USA), low pass filtered at 10 kHz, and digitized at 20 kHz. Access resistance was typically between 20 and 40 MΩ and was compensated at the beginning of every recording and adjusted as required.

### Data Acquisition and Analysis

Data were acquired using pCLAMP 10 software and digitized with a Digidata 1440 (Molecular Devices, San Jose, CA, USA). The analysis was carried out using Clampfit software (Molecular Devices), and results were plotted and statistical analysis performed in Origin 9.1 (OriginLab Corp; Northampton, MA, USA). Whilst using current injection protocols, cells were held at a constant hyperpolarized baseline membrane potential of −60 mV by DC current injection. Cell-attached gap-free recordings were made in voltage-clamp after obtaining a seal of 1 GΩ or tighter with the patch held at 0 mV and no external voltage command.

### Chemicals

DNQX, gabazine, DL-AP5, XE991, muscarine, t-ACPD, CPCCOet, and MPEP were obtained from Tocris Bioscience (Bristol, UK). Potassium gluconate, flufenamic acid (FFA), and the other substances used for preparing the solutions were obtained from Sigma-Aldrich Norway AS (Oslo, Norway). All chemicals tested in the experiments were bath applied at a superfusion rate of ~2 ml min^−1^.

## Results

### Spontaneous Firing in OLM Cells Is Increased by Muscarinic or Metabotropic Glutamate Modulation

**Figure 1:**
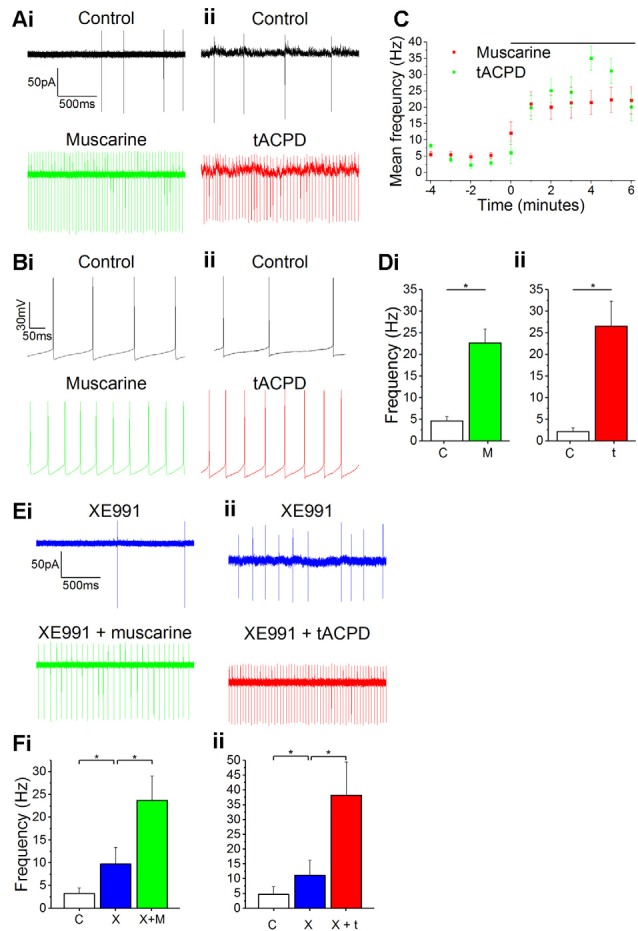
Increased spontaneous firing rate following mAChR/mGluR activation is only partly mediated by M current. **(A,B)** Example traces showing spontaneous action potential firing observed during gap-free recording in control conditions in both cell-attached **(A)** and whole-cell **(B)** patch configurations. The frequency of firing increased in response to the application of either 10 μM muscarine **(Ai,Bi)** or 15 μM t-ACPD **(Aii,Bii)**. **(C)** The rate of spontaneous firing during cell-attached recording in control conditions and the increase in frequency following the application of either muscarine or tACPD (presence of drug indicated by the black bar). Error bars = SEM. *p < 0.05. **(D)** The summary plot of the spontaneous firing frequency change for both muscarine (**Di**; *n* = 5) t-ACPD (**Dii**; *n* = 6). **(E)** Example traces showing an increase in spontaneous firing following the application of either muscarine (**Ei**) or tACPD (**Eii**) in addition to XE991. **(F)** The summary plot of the spontaneous firing frequency change following XE991 application, and application of either muscarine (**Fi**; *n* = 7) or t-ACPD (**Fii**; *n* = 6). Kv7 blockade increased the firing rate from the control and the application of mGluR or mAChR agonists significantly increased the firing rate further. Error bars = SEM. *P < 0.05.

When recording from our genetically labeled OLM cells (*n* = 14) in *Chrna2* transgenic mice, we always observed spontaneous firing of action potentials (3 ± 0.74 Hz) in both cell-attached voltage clamp (Figures 1Ai–ii) and whole-cell current clamp recordings (Figures 1Bi–ii) during control conditions. Such spontaneous firing has previously been reported in OLM cells in acute slice preparations (Leão et al., 2012) and we began by testing whether this firing was modulated by mAChR or mGluR activation. When testing this, we observed a clear increase in the spontaneous action potential frequency during the wash-in of either the mAChR agonist muscarine (10 μM) or the mGluR agonist tACPD (15 μM; Figure 1C). Thus, significant increases in firing rate were seen following application of either muscarine (from 4.7 ± 1.0 Hz to 22.7 ± 3.3 Hz after muscarine application; *n* = 5; paired *t*-test, *P* = 0.05; Figure 1Di) or t-ACPD (from 0.6 ± 0.4 to 23.6 ± 7.1 Hz; *n* = 6; *P* = 0.02; Figure 1Dii).

Both mAChR and mGluR activation are known to reduce the M-type potassium current (*I*_M_, mediated by Kv7 channels) activity (Charpak et al., 1990; Shapiro et al., 2000), and modulation of these channels was found to affect resting membrane potential and spontaneous firing frequency in OLM cells (Lawrence et al., 2006). Therefore, we sought to determine whether the increase in spontaneous firing seen in OLM cells following activation of these receptors was due to their suppression of Kv7 channels. Using the selective Kv7 channel blocker XE991 (10 μM), we observed significantly increased spontaneous firing compared to control conditions (from 4.7 ± 2.6 to 11.1 ± 5 Hz; *n* = 14; *P* = 0.04; Figure 1F). After XE991 application, either muscarine or t-ACPD was added to test whether their effects were occluded by prior Kv7 blockade. However, we observed no signs of such occlusion. Thus, the addition of these agonists was followed by significant additional increases in spike frequencies, increases even greater than the difference observed between control and XE991 conditions. Addition of muscarine increased the spike frequency from 9.7 ± 3.7 to 23.7 ± 5.3 Hz (*n* = 11; *P* = 0.00005; Figure 1Fi), and addition of t-ACPD increased the frequency from 11.1 ± 5.1 to 38.2 ± 11.2 Hz (*n* = 6; *P* = 0.03; Figure 1Fii). This suggests that the increased spontaneous firing seen with activation of mGluR and mAChRs is partly due to blocking of Kv7 channels, but that the increase is largely due to other mechanisms.

### Plateau Potentials in OLM Cells

Lawrence et al. (2006) found plateau potentials in morphologically identified OLM cells following muscarinic activation, and we attempted to verify this finding in our genetically identified OLM cells.

**Figure 2:**
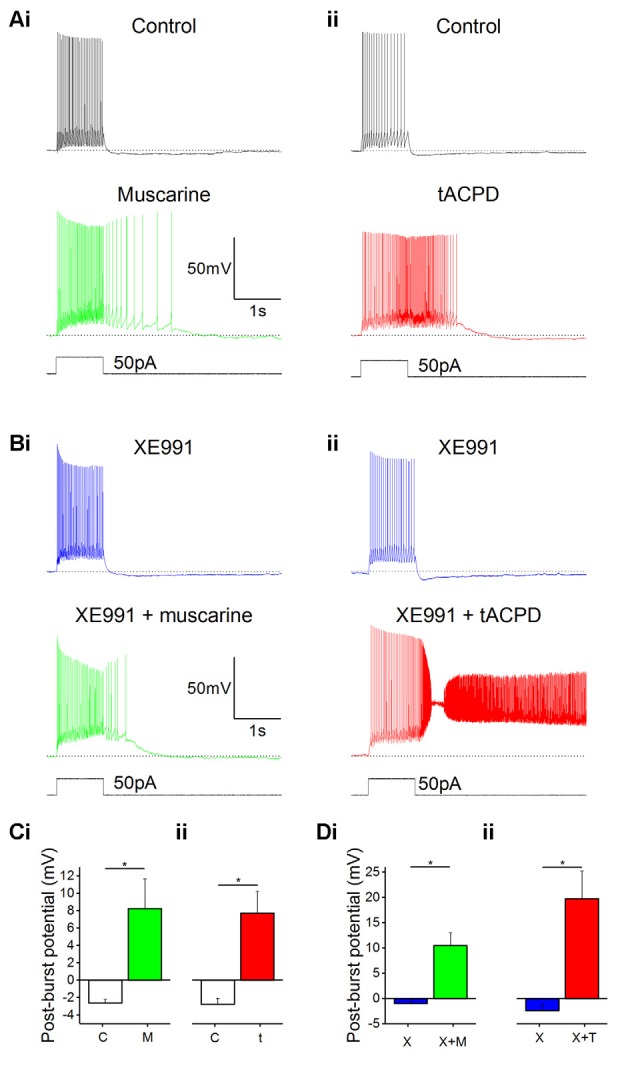
mAChR and mGluR activation induce plateau potentials in oriens-lacunosum moleculare (OLM) cells. **(A)** Example traces showing the onset of a plateau potential following the application of either muscarine **(Ai)** or t-ACPD **(Aii)**. **(B)** Example traces showing that blocking M-current with XE991 did not induce post-pulse spiking or plateau potentials, but these effects could be induced with additional application of muscarine **(Bi)** or tACPD **(Bii)**. The horizontal, dotted lines in **(A,B)** indicate the baseline membrane potential during a 200 ms time window prior to the current pulse onset. **(C)** Summary plots showing the change in post-burst potential from control with either application of either muscarine (**Ci**; *n* = 6) or tACPD (**Cii**; *n* = 7), moving from an afterhyperpolarization (AHP) to an after-depolarization (ADP) in both conditions. **(D)** Summary plots showing the change in post-burst potential in the presence of XE 991 and following the application of either muscarine (**Di**; *n* = 6) or t-ACPD (**Dii**; *n* = 6). Both agonists induced a significant change in the post-pulse potential following in the presence of XE 991. Error bars = SEM. *P < 0.05.

In line with previous reports, we observed that bath application of muscarine (10 μM) led to a change in post-pulse potential following intracellular injection of a 1 s long 50 pA depolarizing current pulse from a −60 mV holding potential: an afterhyperpolarization (AHP) was replaced by a slow depolarizing plateau afterpotential accompanied by continued spiking (Figure 2Ai). The application of tACPD (15 μM) produced a similar effect (Figure 2Aii). Similar to previous studies, we defined the post-burst potential as the difference between the mean membrane potential during a 200 ms time window immediately prior to the current pulse (dotted lines in Figures 2A,B) and the mean membrane potential during a 200 ms time window following 100 ms after the offset of the current pulse injection (Lawrence et al., 2006). The post-pulse potential changed significantly from control following application of either muscarine (from −2.6 ± 0.4 mV to 8.2 ± 3.4 mV; *n* = 6; *P* = 0.02; Figure 2Ci) or tACPD (from −2.8 ± 0.7 mV to 7.7 ± 2.5 mV; *n* = 6; *P* = 0.008; Figure 2Cii). The emergence of plateau potentials caused a dramatic increase in the time taken for the membrane potential to return to baseline following the offset on the current pulse, from 144 ± 19 ms under control conditions, to 716 ± 281 ms following muscarine application, and 1383 ± 401 ms following tACPD application.

### Modulation of the M Current Is not Responsible for the Changes in Post-burst Potential Following Muscarinic or Glutamatergic Metabotropic Modulation

Having observed that both muscarinic or glutamatergic metabotropic receptor activation induce a depolarizing plateau potential in the OLM interneurons, we searched for the underlying ionic mechanism.

*I*_M_, which is reduced by both mAChR and mGluR activation (Brown and Adams, 1980; Halliwell and Adams, 1982; Charpak et al., 1990; Shapiro et al., 2000), is known to contribute to AHPs in mammalian central neurons (Storm, 1989). Thus, a metabotropic suppression of the hyperpolarizing *I*_M_, might possibly cause a depolarizing plateau. To test this hypothesis, we bath-applied the selective *I*_M_ blocker XE991 (Wang et al., 1998) and compared the post-burst potential before and after adding either muscarine (Figures 2Ai,Bi) or tACPD (Figures 2Aii,Bii), in the absence (Figure 2A) or presence (Figure 2B) of 100 μM XE991, in order to determine the contribution of M-current to the post-burst potential. Shallow AHPs were still observed in the presence of XE991 (Figures 2Bi,Bii), and significant changes with emergence of large plateau potentials were still seen (as in XE991-free control conditions) with addition of either muscarine [from −1.0 ± 0.1 mV (AHP) to 10.5 ± 2.6 mV (plateau); *n* = 6; *P* = 0.006; Figure 2Di] or tACPD [from −2.4 ± 1.3 (AHP) to 19.8 ± 5.6 mV (plateau); *n* = 6; *P* = 0.008; Figure 2Dii]. This showed that plateau potentials can still be generated in these cells during blockade of *I*_M_. Therefore, another mechanism besides *I*_M_ modulation is necessary to explain the induction of plateau potentials by mGluR and mAChR activation.

### Calcium Dependence of Glutamatergic and Muscarinic Plateau Potentials

Having excluded the possibility that the depolarizing plateau potentials are merely due to suppression of *I*_M_, we tested other possible mechanisms. Since several neuronal afterpotentials are calcium-dependent, including muscarine-induced plateaus in stratum oriens interneurons (Lawrence et al., 2006), we tested whether reducing either extracellular or intracellular (Ca^2+^) affected the plateau potentials in OLM cells in response to muscarine and tACPD.

**Figure 3:**
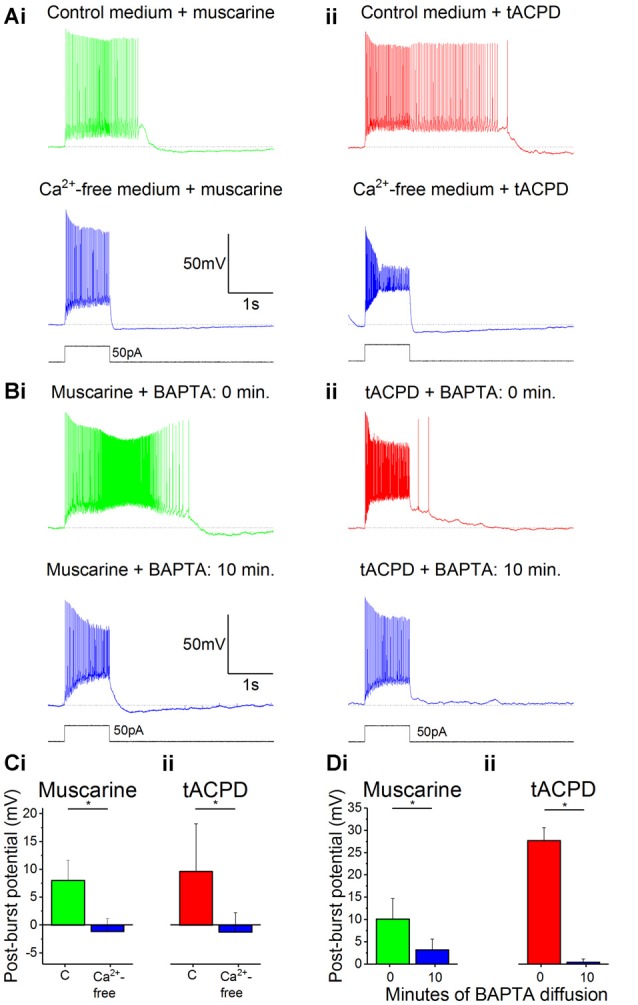
The muscarinic and glutamatergic plateau potentials are calcium-dependent. **(A)** Example traces of the abolition of muscarine- **(Ai)** or tACPD-induced **(Aii)** plateau potentials by switching to a Ca^2+^-free artificial cerebrospinal fluid (aCSF) medium in the continuous presence of agonists. **(B)** Example traces showing the abolition of muscarine- **(Bi)** or tACPD-induced **(Bii)** plateau potentials immediately after (upper traces) and 10 min after break-in (lower traces) when the BAPTA-containing pipette solution became contiguous with the intracellular space. **(C)** Summary plots of the change in post-burst potential in the presence of either muscarine (**Ci**; *n* = 6) or tACPD (**Cii**; *n* = 7) after switching to a Ca^2+^-free medium, showing a move from an ADP to an AHP. **(D)** Summary plots of the change in post-burst potential in the presence of muscarine (**Di**; *n* = 5) or tACPD (**Dii**; *n* = 8) after 10 min of allowing BAPTA to diffuse into the intracellular solution. Error bars = SEM. *P < 0.05.

After switching from our standard control aCSF extracellular medium to a Ca^2+^-free medium in the constant presence of either muscarine (Figure 3Ai) or tACPD (Figure 3Aii), we observed a significant reduction in the post-burst plateau potentials in the presence of either muscarine [from 9.6 ± 8.6 mV (plateau) to −1.2 ± 3.4 mV (AHP); *n* = 7; *P* = 0.02; Figure 3Ci] or tACPD [from 8.0 ± 3.6 mV (plateau) to −1.1 ± 2.3 mV (AHP); *n* = 6; *P* = 0.04; Figure 3Cii], thus tending to move from a plateau to an AHP in both cases.

We next tested the effects of reducing the intracellular calcium level by including 10 mM of the calcium chelator BAPTA in the pipette solution (Storm, 1987). The plateau potentials were observed immediately after break-in (Figure 3Bi, 0 min), but gradually disappeared as BAPTA diffused into the cell from the pipette, still in the continuous presence of muscarine (Figure 3Bi) or tACPD (Figure 3Bii) in the bath. Thus, after 10 min the post burst plateau potential was significantly reduced, both in the presence of muscarine (from 10.1 ± 4.6 to 3.2 ± 2.4 mV; *n* = 5; *P* = 0.04; Figure 3Di) and with tACPD (from 27.7 ± 2.9 to 0.4 ± 0.8 mV; *n* = 8; *P* = 0.00001; Figure 3Dii). The presence of clear AHPs in Ca^2+^-free medium and with BAPTA in at least some cells (Figures 3Ai,Aii, lower panels), indicates that these rather slow AHPs are not caused by Ca^2+^-dependent potassium currents, unlike slow AHPs in many vertebrate principal neurons (Lancaster et al., 2001; Vogalis et al., 2003).

We wanted to ensure that the effects we observed when removing extracellular Ca^2+^ or chelating intracellular Ca^2+^ were not due to a time-dependent “rundown” of the plateau potentials (McQuiston and Madison, 1999). To do this, we performed control experiments where plateau potentials were recorded immediately following the application of the agonist, then again after 15 min later, with no other intervention.

The post-burst potential amplitude did not change after 15 min of recording in the presence of either muscarine (from 10.7 ± 3.1 mV at 0 min to 11.8 ± 3.5 mV at 15 min; *n* = 6; *P* = 0.59; data not shown) or tACPD (from 28.4 ± 1.8 mV at 0 min to 31.1 ± 2.5 mV at 15 min; *n* = 7; *P* = 0.31; data not shown). Therefore, we assume that the reduced amplitude of the plateau potential we observed when (then?) was not the result of a rundown.

### TRPC Channels Mediate Plateau Potentials Induced by mAChRs and mGluRs

**Figure 4:**
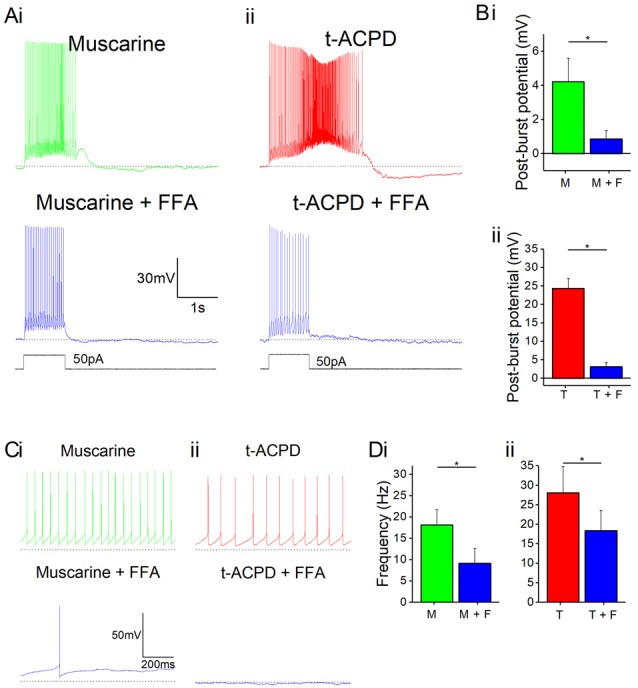
mAChR and mGluR agonist-induced effects can be reversed by a non-specific transient receptor potential (TRP) channel blocker, flufenamic acid (FFA). **(A)** Example traces showing plateau potentials induced (upper traces) by application of 10 μM muscarine **(Ai)** or 15 μM tACPD **(Aii)** are blocked by the addition of the non-specific TRP channel blocker, FFA (50 μM; lower traces). **(B)** Summary plots of post-pulse potential following application of FFA in the presence of either muscarine (**Bi**; *n* = 5) or tACPD (**Bii**; *n* = 4). In both cases, it was significantly reduced. **(C)** Example traces showing spontaneous firing in the presence of muscarine **(Ci)** or tACPD **(Cii)** before (upper trace) and after (lower trace) the addition of FFA. **(D)** Summary plots of the spontaneous firing rate following application of FFA in the presence of either muscarine (**Di**; *n* = 10) or tACPD (**Dii**; *n* = 10). Error bars = SEM. *P < 0.05. The firing rate significantly decreased in the presence of either mGluR or mAChR agonists.

Next, we asked what channel type could underlie the plateau potentials induced by mAChR or mGluR. A plausible candidate mechanism is transient receptor potential C (TRPC) channels, which have been shown to mediate plateau potentials in cortical pyramidal cells (Zhang et al., 2011). To test this idea, we used non-specific TRPC channel blocker FFA following the application of either mAChR or mGluR agonists. Application of 50 μM FFA blocked the plateau potentials induced by 10 μM muscarine (Figure 4Bi) or 15 μM tACPD (Figure 4Aii). In both cases, the post-pulse potentials were significantly reduced by FFA, from 4.2 ± 1.4 to 0.9 ± 0.5 mV after muscarine (*n* = 5; *P* = 0.002; Figure 4Bi), and from 24.9 ± 4.3 to 1.9 ± 1.0 mV after tACPD (*n* = 4; *P* = 0.007; Figure 4Bii).

FFA also reduced the spontaneous firing in the presence of either muscarine (Figure 4Ci) or tACPD (Figure 4Cii). Thus, spontaneous action potential frequency was significantly reduced by FFA both when applied in the presence of either muscarine (from 18.2 ± 3.6 to 9.2 ± 3.5 Hz; *n* = 10; *P* = 0.002; Figure 4Di) or tACPD (from 28.1 ± 6.7 to 18.4 ± 5.2 Hz; *n* = 6; *P* = 0.04; Figure 4Dii). This strongly suggests that metabotropic stimulation of TRPC channels underlie also the mACh and mGluR-induced increase in spontaneous firing, in addition to the evoked plateau potentials, in OLM cells.

### Group I mGluRs Are Responsible for the Glutamatergic Plateau Potentials

**Figure 5:**
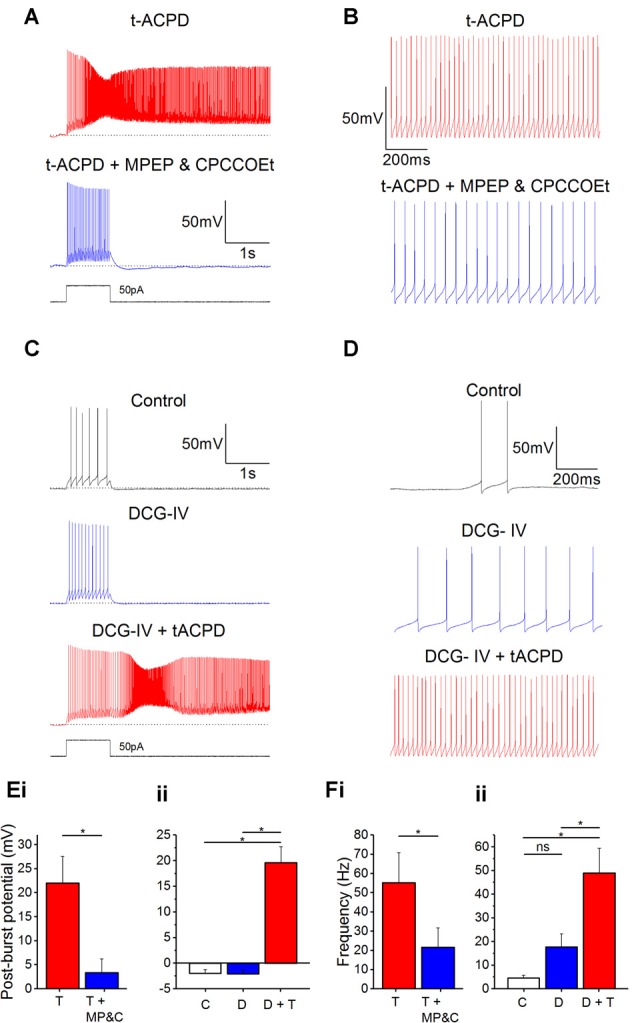
Plateau potentials are evoked by group I but not group II mGluR activation. **(A)** Typical response to 50 pA 1 s current pulse in the presence of 15 μM tACPD, before and after application of the mGlu5R antagonist MPEP and the mGlu1R antagonist CPCCOEt. **(B)** Example traces showing the decrease in spontaneous firing in the presence of t-ACPD following addition of MPEP and CPCCOEt. **(C)** Example traces of plateau potentials following application of the selective Group II mGluR agonist DCG-IV and DCG-IV and tACPD. **(D)** Example traces of spontaneous firing following application of DCG-IV and DCG-IV and tACPD. **(E)** Summary plots of the post-pulse potential in the presence of t-ACPD following the addition of MPEP and CPCCOEt (**i**; *n* = 5) and following DCG-IV application and subsequence tACPD application (**ii**; *n* = 5). **(F)** Summary plots of spontaneous firing frequency in the presence of tACPD following the application of MPEP and CPCCOEt (**i**; *n* = 4), and in response to DCG-IV and following sequential application of tACPD (**ii**; *n* = 5). Error bars = SEM. *P < 0.05.

tACPD is an agonist at both group I (mGlu1 and mGlu5) and group II mGluRs (mGlu2 and mGlu3; Pin and Duvoisin, 1995). Hence, we sought to determine if activation of one group was more responsible than the other for plateau potential induction and increased spontaneous firing. By applying antagonists for group I receptor subtypes mGlu1 Rs (CPCCOEt; 100 μM) and mGlu5 Rs (MPEP; 60 μM) in the presence of tACPD, we observed that plateau potentials were abolished (Figure 5A) and the post-burst potential significantly decreased (from 22 ± 5.5 to 3.4 ± 2.9 mV; *n* = 6; *P* = 0.02; Figure 5Ei).

These mGlu1 and mGlu5 R antagonists also produced a change in the spontaneous firing in the presence of tACPD (Figure 5B). Firing frequency was significantly reduced (from 55.2 ± 15.6 to 21.7 ± 10 Hz; *n* = 4; *P* = 0.02; Figure 5Fi), but did not reach the previously established levels of spontaneous firing in control conditions (0.58 ± 0.37 Hz; Figure 1D).

These results indicated that the tACPD-induced plateau is wholly dependent on group I mGluR activation whilst the tACPD induced increase in spontaneous firing is at least partially dependent on the group I mGluRs. To test the impact of group II mGluRs, we added DCG-IV (10 μM), a group II-selective agonist (Brabet et al., 1998; Mateo and Porter, 2007), in the absence of tACPD. The post-burst potential did not change with application of DCG-IV (from −2.0 ± 0.7 to −2.1 ± 0.8 mV; *n* = 5; *P* = 0.65; Figure 5C) but subsequent application of tACPD in the same cells elicited a significant increase (from −2.1 ± 0.8 to 19.6 ± 3.1 mV; *n* = 5; *P* = 0.03; Figure 5C) as expected from the previous experiments (Figures 1–4).

Spontaneous firing following application of DCG-IV (Figure 5D), did not change significantly (from 4.5 ± 1.3 to 17.7 ± 5.6 Hz; *n* = 7; *P* = 0.09; Figure 5Fii) whilst the increases in frequency caused by application of tACPD were shown to be significant (from 17.7 ± 5.6 to 49 ± 10.4 Hz; *n* = 7; *P* = 0.03; Figure 5Fii). These results strongly suggest that Group I but not Group II mGluRs underlie the metabotropic glutamatergic enhancement of both spontaneous firing and evoked plateau potentials in OLM cells.

### Non-linear Synergistic Induction of Plateau Potentials by mAChR and mGluR Co-activation

**Figure 6:**
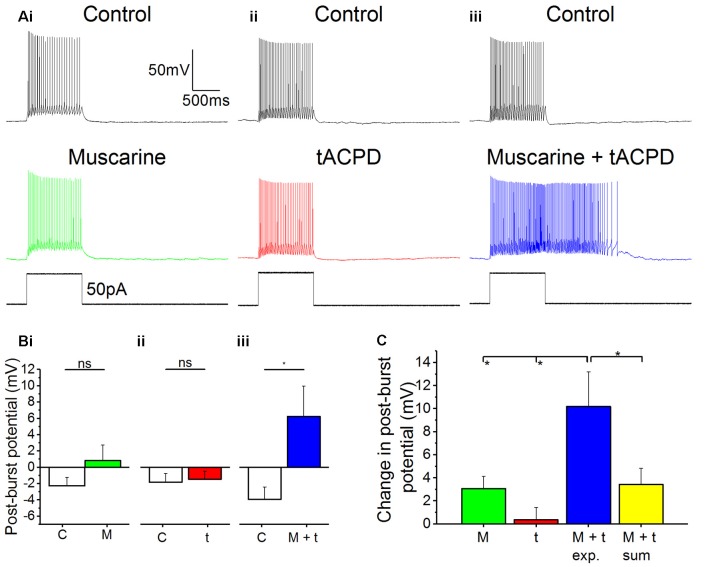
Synergistic effects on post-pulse potential by coapplication of low doses of muscarine and tACPD. **(A)** Typical responses of OLM cells to a 50 pA 1 s current pulse before and after application of either a low dose muscarine (**Ai**; 4 μM; *n* = 8), a low dose tACPD (**Aii**; 4 μM; *n* = 8), or both combined (**Aiii**; 4 μM + 4 μM; *n* = 6). **(B)** Summary plots show that no significant change in post-burst potential was seen with application of a low dose of muscarine (**Bi**; *n* = 8) or tACPD (**Bii**; *n* = 8), whereas a significant increase was seen following application of a low doses of both drugs simultaneously (**Biii**; *n* = 6), converting the post-burst potential from and AHP to ADP. **(C)** The Summary plot for change in post-pulse potential, in all experimental conditions and also *post hoc* linear sum (yellow column). The linear sum was calculated from eight randomly selected pairs of independent measures of single low dose drug administration. Error bars = SEM. *P < 0.05; ns, not significantly different, *P* > 0.05.

In many *in vivo* situations, such as in awake, behaving animals, it is likely that both cholinergic and glutamatergic metabotropic neuromodulation occur simultaneously in the same cell, activated by distinct afferents *via* their respective receptors. It is then functionally important whether these converging forms of metabotropic neuromodulation are occluding each other, or are linearly additive, or perhaps non-linearly synergistic. To explore this issue, we used low concentrations of the mAChR and mGluR agonists (4 μM muscarine; 4 μM tACPD), applied both individually and simultaneously. We observed no plateau potentials after adding either the low dose of muscarine alone (Figure 6Ai) or after adding the low dose of tACPD alone (Figure 6Aii). In contrast, simultaneous application of the same low doses of both these agonists led to an emergence of a plateau (Figure 6Aiii). This was confirmed by the lack of significant change in post-burst potential in the presence of either low dose muscarine (from −2.3 ± 1 to 0.8 ± 1.9 mV; *n* = 8; *P* = 0.1; Figure 6Bi) or low dose tACPD (from −1.8 ± 1.1 to −1.5 ± 1 mV; *n* = 8; *P* = 0.74; Figure 6Bii), whereas we observed a significant increase in the presence of both agonists (from −3.9 ± 1.5 to 6.2 ± 3.7 mV; *n* = 6; *P* = 0.01; Figure 6Biii), which caused the transformation of the post-burst potential from an AHP to an ADP.

We wanted to determine whether the effect of combining low doses of both drugs experimentally was any different from what might be expected from the addition of effects seen in the presence of individual drugs alone. To do this we summed eight randomly selected pairs of measurements from independent drug administrations and compared them with the values obtained during experimental recordings in the presence of both agonists.

The mean change in post-burst potential in the presence of both muscarine and tACPD (Figure 6C; 10.2 ± 3 mV; *n* = 8) was significantly increased from control when compared to the values obtained in the presence of either just muscarine (3.1 ± 1.1 mV; *n* = 8; *P* = 0.02) or just tACPD (0.4 ± 1.13 mV; *n* = 8; *P* = 0.004). When the low concentrations of agonists were applied together, the post-burst potential was significantly greater than the sum of the effects of low concentrations of agonists applied separately (10.2 ± 3 simultaneous vs. 3.4 ± 1.4 mV summed; summed *n* = 8; *P* = 0.04). These results demonstrate that the cholinergic and glutamatergic forms of metabotropic neuromodulation are not only converging but are also acting in a non-linear, synergistic manner in OLM cells.

## Discussion

Our results show that the intrinsic activity and electrophysiological properties of OLM cells are strongly modulated by glutamatergic and cholinergic neuromodulatory systems, causing these neurons to both increase their spontaneous firing rate and produce plateau potentials and spiking output that can outlast the duration of the depolarizing stimuli by several seconds. We have identified the metabotropic receptor types responsible for the modulation and the downstream signaling targets of these receptors which lead to the modulatory changes. Finally, we observed pronounced synergy between the cholinergic and glutamatergic modulatory systems. Taken together, these results suggest that these cells may contribute significantly to switching of the information flow in the CA1 circuit, within and between functional modes of the hippocampus, and potentially contributing to switching between different brain states.

### Depolarizing Plateau Potentials

mAChR agonists have previously been shown to induce plateau potentials in morphologically identified putative-OLM cells (Lawrence et al., 2006), and we demonstrate here that this occurs in genetically identified Chrna2-expressing OLM cells.

Plateau potentials are accompanied not just by increased spiking but also by spikes bursts (Takahashi and Magee, 2009). These bursts may signal to downstream targets of OLM cells that a specific convergence of signals of distinct origins or transmitter systems has occurred (Zeldenrust et al., 2018).

Plateau potentials in cortical cell dendrites are known to facilitate LTP induction during sensory processing (Gambino et al., 2014), as the prolonged depolarization and spiking associated with plateaus increases the window of opportunity for Hebbian pairing. Recently, Bittner et al. (2017) discovered that place fields in hippocampal area CA1 are produced by a form of synaptic potentiation that is notably different from Hebbian plasticity. This *behavioral time scale synaptic plasticity* (BTSP) can store behavioral sequences within synaptic weights to produce predictive place cell activity and could be mimicked in brain slices by pairings of presynaptic activity and calcium plateau potentials, thus producing large potentiation with a seconds-long time course (Bittner et al., 2017). The seconds-long plateau potentials occurring in OLM cells under wake-like conditions (Figures 1–5) may be suited to regulate BTSP in the pyramidal cells. If a BTSP-like plasticity can also occur in the OLM interneurons, in addition to the excitatory cells where it was already observed, this may substantially expand the possibilities for plasticity and tuning of hippocampal and other cortical microcircuits during learning.

### Modulation of Spontaneous Firing

We found that OLM cells increased their rate of spontaneous firing in response to agonists of either metabotropic acetylcholine receptors or metabotropic glutamate receptors. Depolarization and increased frequency of spontaneous firing has been shown in multiple cell types in response to mAChR (Smetana et al., 2007; Dennis et al., 2016) or mGluR (Chuang et al., 2000; Young et al., 2004) activation. In OLM cells, cholinergic and glutamatergic boosting of firing rate will enhance the tonic inhibitory control over their postsynaptic targets: increasing inhibition to the distal apical dendritic compartment of pyramidal cells (Sik et al., 1995) whilst disinhibiting the proximal apical dendrite (Leão et al., 2012). This will prioritize the proximal Schaffer collateral CA3 inputs over the distal perforant path EC inputs. OLM cell activity has been suggested as a mechanism for suppressing current, incoming sensory information from the EC and thereby instead promoting the processing of information from the CA3, where learned patterns are stored in the autoassociative CA3 network (Leão et al., 2012). This may suggest that conditions that increase OLM cell activity may tend to promote memory retrieval and impair memory acquisition (Gold, 2003).

Increased OLM cell activity in response to mAChR activation could contribute to the impact of the medial septum, which provides cholinergic inputs to the hippocampus during wakefulness and arousal, during learning and memory. For example, inactivation of the medial septum by lidocaine was found to impair new memory formation and memory retrieval in rats (Koenig et al., 2008) and medial septum inactivation also reduced memory persistence (Parfitt et al., 2012). Further work is needed to further elucidate the extent to which the promotion of memory and learning *via* septal cholinergic inputs is dependent on OLM cells.

### Receptor Types and Intracellular Signaling Pathways

OLM cells receive facilitating excitatory input, suggesting they have “class 2” glutamatergic synapses which contain both ionotropic (iGluRs) and metabotropic (mGluRs) glutamate receptors (Viaene et al., 2013). These cells also express nicotinic AChRs (Nakauchi et al., 2007), and of course iGluRs. This suggests that the depolarization mediated by glutamatergic or cholinergic input *in vivo* will occur *via* both ionotropic and metabotropic receptors, and hence the resulting depolarization will be even greater than we observed in this study.

The longer duration of metabotropic compared to ionotropic receptor-mediated postsynaptic effects can sustain repetitive firing that persists long after the decay of the fast postsynaptic events.

### mAChR Subtypes

Acetylcholine-dependent plateaus in OLM cells were shown to be mediated by type 1 and type 3 mAChRs (Lawrence et al., 2006), both of which associate with G-proteins of the G_q_ type (Hulme et al., 1990). We found that the mGluR subtypes responsible for glutamate-dependent plateaus belong to the group I mGluRs, which includes mGluR_1_ and mGluR_5_, both of which also associate with G_q_ (Swanson et al., 2005). This indicates that G_q_ is probably responsible for inducing plateau potentials in OLM cells. Interestingly, in spinal motoneurons, G_q_-coupled 5-HT2 receptors also promote plateau potentials (Perrier and Hounsgaard, 2003), further suggesting the crucial involvement of G_q_ across neuron types, and that other G_q_-coupled receptors present in OLM cells may also contribute to plateau potentials.

### mGluR Subtypes

Previous studies in pyramidal neurons have shown that group I mGluR activation leads to depolarization (Chuang et al., 2000; Young et al., 2004), whereas group II activation leads to hyperpolarization (Cox and Sherman, 1999). Therefore, the depolarization caused by the pan-mGluR agonist t-ACPD (Figures 1, 2, 5) suggests that group I mGluRs are likely to be the predominant mGluR subtype group underlying this type of modulation in OLM cells. Antagonists at the group I mGluRs reduced the spontaneous firing frequency that had previously been increased by a pan-mGluR agonist, suggesting that group I subtype receptors also modulate the spontaneous firing of OLM cells.

Whilst the increase in spontaneous firing in response to group II mGluR agonist DCG-IV did not reach statistical significance in our experiments (*P* = 0.088), an increase in the mean firing rate was observed as opposed to the hyperpolarization observed in response to group II agonists in other cells (Hermes and Renaud, 2011; Bocchio et al., 2019).

### TRP Channels and Calcium-Dependence

Earlier work has shown that TRP channels are activated by mGluRs (Gee et al., 2003; Ben-Mabrouk et al., 2012) and mAChRs (Delmas et al., 2002; Moran et al., 2011) in various cell types. We saw that the increase in spontaneous firing caused by mAChR or mGluR activation was reduced in the presence of the TRP-channel blocker FFA, suggesting that TRP channels contribute to regulating the prevailing membrane potential and spontaneous firing in these cells during cholinergic or glutamatergic modulation.

FFA also abolished the plateau potentials, indicating that they depended on TRP channels. We found that the plateau potentials were also suppressed by Ca^2+^-free medium and by intracellular BAPTA, indicating a dependence on raised intracellular free Ca^2+^. It is possible that Ca^2+^ influx through TRP channels may also contribute directly to the plateau depolarizations, as many TRP channels have a significant Ca^2+^ permeability (Owsianik et al., 2006; Venkatachalam and Montell, 2007). Voltage-gated Ca^2+^ (Ca_v_) channels may also contribute directly to the plateau potentials in OLM cells, like in some other neurons (Williams and Fletcher, 2019), but are unlikely to be the main mechanism, since the plateaus were abolished by FFA and BAPTA, but Ca_v_ channels may still contribute indirectly, by conducting Ca^2+^ influx that trigger TRP channels. The plateau potentials we observed were NMDA-R-independent (resistant to NMDA-R-blockade), as expected since CA1 OLM cells do not express NMDA receptors (Oren et al., 2009). This is in contrast to some plateaus in other cell types that depend on regenerative activation of NMDA receptor channels (Gambino et al., 2014; Palmer et al., 2014). Enhanced TRP channel activity has been demonstrated in response to PKC activation (Mandadi et al., 2011), itself a consequence of activation of Gq-coupled G-protein coupled receptors (GPCRs) such as mAChR1/3 and mGluR1/5, suggesting that this signaling pathway may underlie the initiation of plateau potentials. Another possible mechanism is Ca^2+^ mediated activation of TRP channels (Hasan and Zhang, 2018) by Ca^2+^ release from internal stores, which has been shown to be involved in plateau potentials in some cells types (Mejia-Gervacio et al., 2004). Future work should address the potential roles of these and other mechanisms in the generation of plateau potentials in OLM cells.

TRP-dependent plateau potentials have been observed in many other cell types (Egorov et al., 2002; Tahvildari et al., 2007; Zhang et al., 2011; Bouhadfane et al., 2013). In addition, voltage-gated calcium (Ca_V_) channels of the R- (Park and Spruston, 2012; Williams and Fletcher, 2019), N- (Wong et al., 2013), and L-types (Lo and Erzurumlu, 2002; Simon et al., 2003), or NMDA receptor channels, have been shown to generate Ca^2+^-dependent plateau potentials in different cell types. This suggests that plateau potentials represent a conserved response feature implemented by different ion channel types in multiple cell types.

From the present study, we cannot determine precisely which member(s) of the TRP channel family are responsible for the observed plateau potentials. Based on known sensitivities to FFA of a range of TRP channels, combined with the FFA concentration that we saw was effective, some candidates can be suggested based on the concentration of FFA that we used and we saw to be effective. According to Guinamard et al. (2013), TRPC6, TRPM5, TRPM3, TRPC5, TRPV4 and TRPC4 have an IC50 at around the FFA concentration we used in this study, and are all expressed in the mouse hippocampus (Kunert-Keil et al., 2006). Since specific pharmacological blockers are available for only a few TRPC family members, future identification of the exact subtype would most likely require a transgenic knockout approach.

### M Channels

In addition to TRP channels, we also tested whether M-type potassium current (*I*_M_) might also contribute to the increase in spontaneous firing or plateau potentials caused by mAChR or mGluR, since *I*_M_ known to be suppressed by both mAChR and mGluR activation (Brown and Adams, 1980; Halliwell and Adams, 1982; Charpak et al., 1990; Shapiro et al., 2000). Whilst we saw some increase in spontaneous firing in response to blocking the *I*_M_, as previously observed in other cell types (Brown and Adams, 1980; Halliwell and Adams, 1982; Shah et al., 2008), subsequent addition of either mAChR or mGluR agonists elicited a much larger, further increase in firing frequency increase. The magnitude of this increase suggests that the mAChR or mGluR activation-dependent increase in spontaneous firing rate is mediated primarily through another mechanism, such as the TRP channels (above), although parallel modulation of *I*_M_ may also play a role.

### Synergy

Plateau potential generation can result from the convergence of spatially segregated inputs. For example, Larkum et al. (1999) showed that neocortical pyramidal neurons can associate inputs arriving at different cortical layers to trigger dendritic calcium spikes; and Takahashi and Magee (2009) showed that CA1 pyramidal cells receiving simultaneous glutamatergic inputs from CA3 and EC exhibited NMDA-R-dependent plateau potentials in their distal dendrites. The latter study also showed that the summation of inputs was multiplicative, suggesting that non-linear summation may be a characteristic feature of plateau potential generation, whether they are dependent on ionotropic receptors, or metabotropic receptors as our data shows.

Park and Spruston (2012) showed synergistic effects on post-burst ADPs following mGluR and mAChR activation in CA1 pyramidal cells. Whilst these lasting depolarizations were also non-linear, they were not accompanied by additional spiking. This may be because their evoked ADPs followed trains of only five spikes, whilst our current steps generated many more, and the size of the ADP/plateau potential is probably related to the number of preceding spikes (Kodirov et al., 2016; Nishimura et al., 2018).

Our experiments did not address the mechanism whereby supra-linear synergy arises. A possible mechanism to explain this synergy may result from potential dimerization of mGluRs and mAChRs. Some GPCRs can form multimeric complexes wherein agonist binding at one receptor induces, through protein-protein interactions, allosteric changes to neighboring receptors (Rozenfeld and Devi, 2010). This can lead to changes in function such as enhanced affinity for agonists (Gomes et al., 2000), altered probability of desensitization (Pfeiffer et al., 2002), and enhanced ability to activate signaling cascades *via* their G-proteins (Jordan et al., 2003). Such a mechanism is attractive for explaining the synergy we observe as it implies that ligand binding at one receptor in a heteromer will increase the affinity for or response to ligands of the other receptor type in the heteromer. Such an effect is dependent on the ability of mGluRs and mAChRs to form heteromers, and future research is needed to test whether they can do so in OLM cells.

## Conclusion

Overall, this study demonstrates that metabotropic glutamate or acetylcholine receptor activation can modulate intrinsic excitability and response properties of Chrna2 positive OLM cells in the CA1 field of the rodent hippocampus thus both enhancing their spontaneous firing rate and promoting long-lasting, depolarization-evoked plateau potentials. Our results also suggest that OLM cells respond as coincidence detectors of cortical/hippocampal glutamatergic and septal cholinergic input, resulting in supra-linear summation and strongly enhanced output to its targets. These cells may thus play an important role in regulating circuit functions according to modulatory states, and in controlling input priority within the hippocampus during memory encoding, and recall functions.

## Data Availability Statement

Datasets are available on request: the raw data supporting the conclusions of this manuscript will be made available by the authors, without undue reservation, to any qualified researcher.

## Ethics Statement

All animal procedures were approved by the responsible veterinarian of the institute, in accordance with the statue regulating animal experimentation (Norwegian Ministry of Agriculture, 1996). Ethical review and approval by an ethics committee was not required for this animal study, because such a specific ethical approval is not required for individual studies utilizing only* ex vivo* experiments.

## Author Contributions

JS and NH-V initiated the study, wrote the manuscript and revised the manuscript. NH-V conducted the experiments and analyzed the data.

## Conflict of Interest

The authors declare that the research was conducted in the absence of any commercial or financial relationships that could be construed as a potential conflict of interest.
